# Smart bandwidth allocation for next generation networks adopting software-defined network approach

**DOI:** 10.1016/j.dib.2018.08.091

**Published:** 2018-08-30

**Authors:** Ayoub Bahnasse, Fatima Ezzahraa Louhab, Hafsa Ait Oulahyane, Mohamed Talea, Assia Bakali

**Affiliations:** a Lab LTI, Faculty of Sciences Ben M׳SIK, University Hassan II of Casablanca, Morocco; b Naval Royal School, Casablanca, Morocco

## Abstract

This data article contains information on a new intelligent bandwidth allocation model for future network (Smart Allocation). The included data describe the topology of the network testbed and the obtained results. Obtained data show the effectiveness of the proposed model in comparison with the MAM and RDM bandwidth allocation models. In relation to the performances evaluation, a variety of flows are used such as: voice over IP (VoIP), video, HTTP, and Internet Control Message Protocol (ICMP). The evaluation criteria are: VoIP latency and jitter, Peak Signal to Noise Ratio (PSNR) video, retransmission video, goodput, HTTP response page, and the Round-Trip Time (RTT) ICMP delay. The presented data are extracted based on simulation.

**Specifications Table**

**Table:** 

Subject area	*Network Management, Communication Network Architecture, Network Layer, Network Design.*
More specific subject area	*Quality of Service, Software-Defined Network, Next Generation Networks.*
Type of data	*Table, graph, figure*
How data was acquired	*GNS3 simulator, Cisco XR, Cisco 7200 IOS.*
Data format	*Analyzed*
Experimental factors	*We measured the quality of communication by comparing the Smart Allocation model with RDM and MAM.*
Experimental features	*The evaluation is performed by increasing the packets load from 256 bytes to 1024 bytes. The quality of VoIP, the video distortion, and the response time of the HTTP and Internet Control Message Protocol (ICMP) applications were measured for each load.*
Data source location	*LTI Laboratory*
Data accessibility	
Related research article	*Novel SDN Architecture for Smart MPLS Traffic Engineering-DiffServ Aware Management. Future Generation Computer Systems Journal. In press*

**Value of the data**

• Dynamic bandwidth allocation is one of the major concerns in the networking and telecommunications sector.
• The proposed model allows equitable distribution of bandwidth resources over different flows with different priorities.
• The proposed model is tested for the next generation computer networks. It can be deployed in industrial networks.
• These simulation data can be used as references for future work related to adaptive bandwidth management.
• The proposed model is deployed at a controller; this allows researchers in the Software-Defined Network (SDN) axis to adopt it in order to optimize the performance of their networks.

## 1 Data

With the emergence of new flows (real-time, streaming, gaming, transactional, and file exchange), optimal bandwidth management has become one of the major concerns for both the telecommunication industry actors and the researchers. The Quality of Service (QoS) has become a necessity in the Next Generation Networks (NGN). Several algorithms have been proposed for dynamic bandwidth management while offering clients the solicited QoS level and guaranteeing operators the optimal use of their network infrastructures. The Maximum Allocation Model (MAM) [1] used to distribute in a fixed and absolute way the bandwidth between the different flows. Among the major limitations is the inefficient exploitation of the network resources, which means, even if no flow is present, its bandwidth cannot be temporarily re-allocated to others. The Russian Doll Model (RDM) [2] allows to correct this limit, allowing a bandwidth allocation of a flow for the benefit of another. Except that this allocation can be made just from a low priority flow to another high priority flow and not the reverse. This in fact can condemn the low priority traffic; it cannot be executed in the network. The AllocTC-Sharing [3] is a solution for allowing an allocation in both directions (from lower priority to higher priority and vice versa). However, the models mentioned above take into consideration the bandwidth consumption rate to make the adaptation. The Smart Allocation model makes it possible to take into consideration other criteria related to flows, for example latency, loss rate, and retransmission. The efficiency evaluation of a model must be carried out by increasing the load and varying the flow nature.

## 2 Experimental design, materials, and methods

**Fig. 1:**
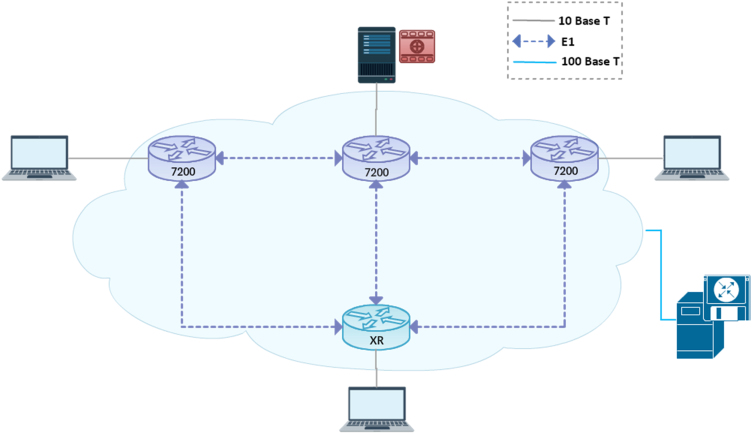
The experiment architecture.

Fig. 1 illustrates the design of the experiment architecture, made under the GNS3 simulator.

The materials used in the experiment are the Cisco XR and Cisco 7200 IOS routers. The Smart Allocation model was deployed on a centralized controller, connected to routers through a 100 Megabit UTP link. The server contains a video sequence of 720 pixels’ resolution. This video will be broadcast to the users to measure the quality of the video in the three bandwidth adaptation models.

The experimental data shown in Table 1 includes the following metrics:

• VoIP latency: the delay between sending a packet and receiving it.
• VoIP jitter: the duration between the sending of two successive packets.
• Page response time: the session opening delay and the delivery of all web content.
• Round-Trip Time (RTT): the delay since sending an ICMP packet to the receipt acknowledgment.

**Table 1:** VoIP latency, VoIP jitter, HTTP response delay, and RTT ICMP.

**Packet Size (bytes)**	**VoIP Latency (ms)**			**VoIP Jitter (ms)**			**HTTP Response Page (ms)**			**ICMP RTT (ms)**		
	**Smart allocation**	**RDM**	**MAM**	**Smart allocation**	**RDM**	**MAM**	**Smart allocation**	**RDM**	**MAM**	**Smart allocation**	**RDM**	**MAM**
**256**	184	204	220	11	19	19	620	620	344	60	64	60
**367**	196	210	218	14	36	40	620	620	400	90	100	120
**512**	208	225	238	17	60	64	650	650	556	130	140	150
**768**	224	244	268	18	65	136	684	684	668	148	180	176
**1024**	244	256	357	50	80	148	750	750	770	190	220	200

The values are represented in milliseconds. The evaluation methodology consists of activating IP SLA probes on routers and programming fully meshed communications. The results were obtained by repeating the same measures ten times in order to ensure the relevance of the obtained results. The parameters of the data used in the experiments are: the G.711 alaw codec for VoIP, version 1.1 of the HTTP protocol, and ICMP Echo type with the Don’t Fragment (DF) flag set.

**Fig. 2:**
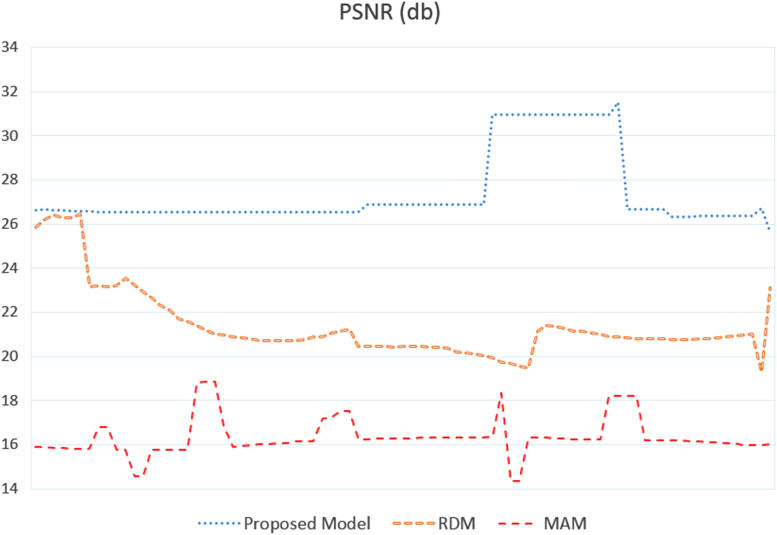
PSNR in dB of the video in the three models: Smart Alloc, RDM, and MAM.

We cannot talk about the QoS without dealing with the video quality. The data in Fig. 2 illustrates the Peak Signal to Noise Ratio (PSNR) of a video sequence intercepted by the Smart Allocation, RDM, and MAM models. These values are obtained from the MSU Video Quality Measurement Tool. The evaluation methodology consists of comparing the quality of the video intercepted by the destination, based on a high-quality file stored in the server. The attached CSV file contains the numeric values used to generate this graph.

**Fig. 3:**
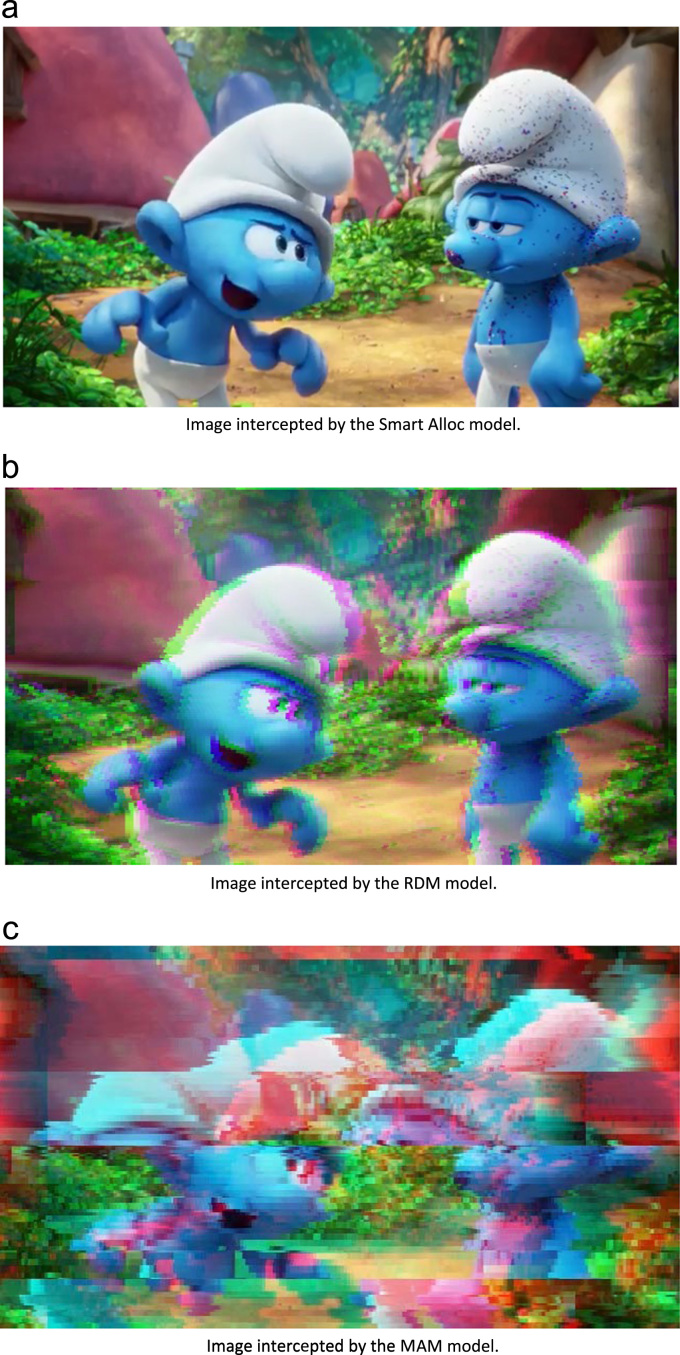
Qualitative evaluation of the video data.

In order to qualitatively evaluate the data, Fig. 3 illustrates a microscopic capture of a video sequence number 87. The Fig. 3a illustrates the image quality with the Smart Allocation model, Fig. 3b with RDM, and Fig. 3c with MAM. The lines represent the glitches and distortions.

**Fig. 4:**
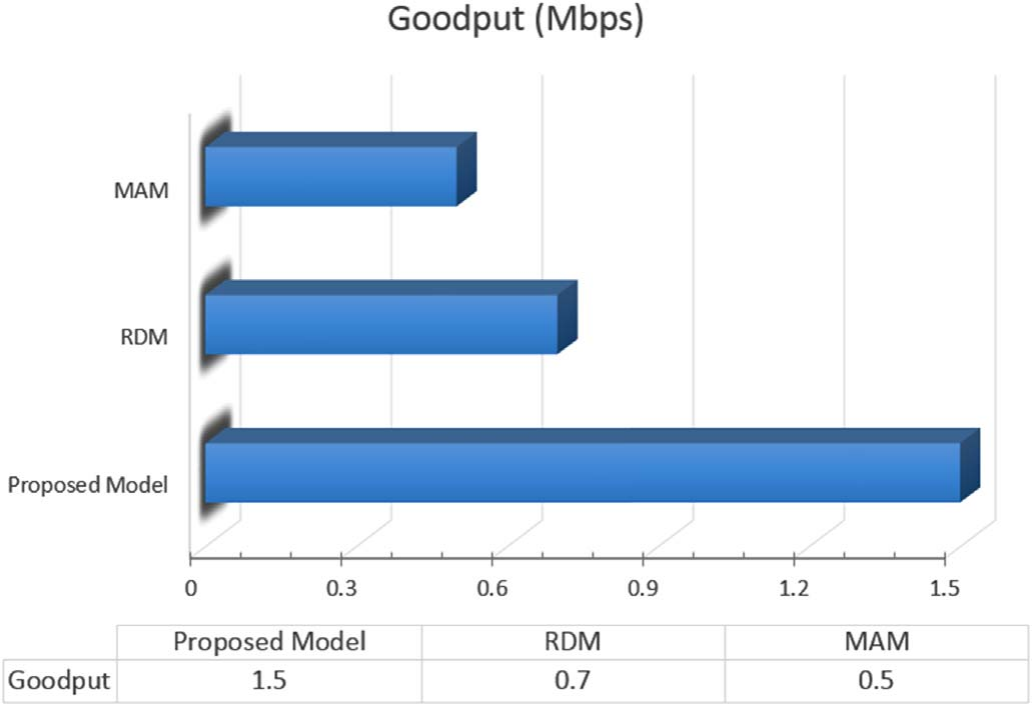
Video goodput data for the Smart Alloc, RDM, and MAM models.

Other data was generated to compare the QoS adaptation models; we mention the goodput and the retransmission. The goodput means the amount of information received by the application layer of the OSI model without including the size of the lower layer headers. The higher the goodput data, the better the quality of the intercepted video. Fig. 4 illustrates the goodput data (in megabits per second) of the three models for the same video sequence used to measure the above criteria.

**Fig. 5:**
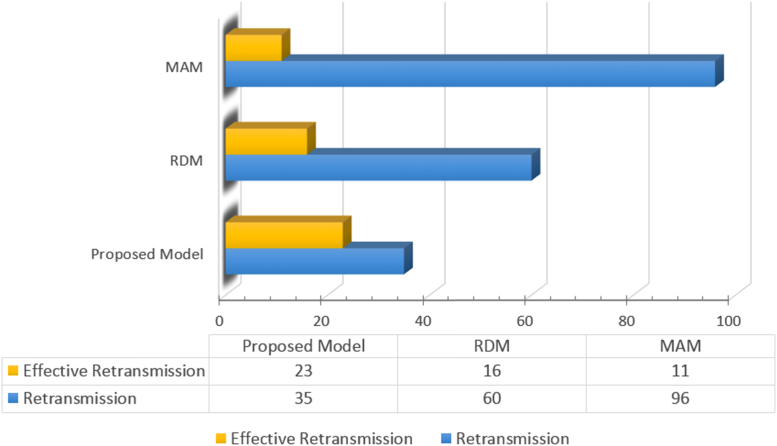
Video retransmission data for the Smart Alloc, RDM, and MAM models.

The packets retransmission is one of the factors that influence the transmissions quality in general and specifically the video quality. Fig. 5 illustrates the retransmissions number in the three models of the same video sequence.

The effective retransmission represents the number of packets successfully retransmitted.
